# Vincamine Modulates the Effect of Pantoprazole in Renal Ischemia/Reperfusion Injury by Attenuating MAPK and Apoptosis Signaling Pathways

**DOI:** 10.3390/molecules27041383

**Published:** 2022-02-18

**Authors:** Michael A. Fawzy, Sherif A. Maher, Mahmoud A. El-Rehany, Nermeen N. Welson, Nisreen K. A. Albezrah, Gaber El-Saber Batiha, Moustafa Fathy

**Affiliations:** 1 Department of Biochemistry, Faculty of Pharmacy, Minia University, Minia 61519, Egypt; michael.fawzy777@mu.edu.eg; 2 Department of Biochemistry, Faculty of Pharmacy, Deraya University, Minia 61111, Egypt; sherif.ali@deraya.edu.eg (S.A.M.); mahmoud.elrehany@deraya.edu.eg (M.A.E.-R.); 3 Department of Forensic Medicine and Clinical Toxicology, Faculty of Medicine, Beni-Suef University, Beni-Suef 62511, Egypt; nermeenwelson@med.bsu.edu.eg; 4 Department of Obstetrics and Gynecology, College of Medicine, Taif University, Taif 21944, Saudi Arabia; dr.nisreen@tu.edu.sa; 5 Department of Pharmacology and Therapeutics, Faculty of Veterinary Medicine, Damanhour University, Damanhour 22511, Egypt; dr_gaber_batiha@vetmed.dmu.edu.eg; 6 Department of Regenerative Medicine, Graduate School of Medicine and Pharmaceutical Sciences, University of Toyama, Toyama 930-0194, Japan

**Keywords:** vincamine, pantoprazole, renal ischemia/reperfusion injury, ROS, MAPK, apoptosis

## Abstract

Pantoprazole has an antioxidant function against reactive oxygen species (ROS). Vincamine, a herbal candidate, is an indole alkaloid of clinical use against brain sclerosis. The aim of the present experiment is to evaluate, on a molecular level for the first time, the value of vincamine in addition to pantoprazole in treating experimentally induced renal ischemia/reperfusion injury (IRI). One-hundred-and-twenty-eight healthy male Wistar albino rats were included. Serum creatinine, blood urea nitrogen, and malondialdehyde levels were assessed. ELISA was used to estimate the pro-inflammatory cytokines. The expression of *Bcl-2* and *Bax* genes was assessed by quantitative real-time PCR. ERK1/2, JNK1/2, p38, cleaved caspase-3, and NF-κB proteins expressions were estimated using western blot assay. The kidneys were also histopathologically studied. The IRI resulted in impaired cellular functions with increased creatinine, urea nitrogen, malondialdehyde, TNF-α, IL-6, and IL-1β serum levels, and up-regulated NF-ĸB, JNK1/2, ERK1/2, p38, and cleaved caspase-3 proteins. Furthermore, it down-regulated the expression of the *Bcl-2* gene and upregulated the *Bax* gene. The treatment with vincamine, in addition to pantoprazole multiple doses, significantly alleviated the biochemical and histopathological changes more than pantoprazole or vincamine alone, whether the dose is single or multiple, declaring their synergistic effect. In conclusion, vincamine with pantoprazole multiple doses mitigated the renal IRI through the inhibition of apoptosis, attenuation of the extracellular signaling pathways through proinflammatory cytokines’ levels, and suppression of the MAPK (ERK1/2, JNK, p38)–NF-κB intracellular signaling pathway.

## 1. Introduction

Renal Ischemia-reperfusion injury (IRI) is a complex disease resulting with transplantation and vascular surgery. Many scientists reported renal IRI as the primary cause of acute renal failure (ARF) [1,2]. Despite the several hypotheses suggested to clarify the pathogenesis of IRI, special attention was centered on the function of reactive oxygen species (ROS) as superoxide radical, hydrogen peroxide, and hydroxyl radical [3]. While all molecules are prone to ROS injury, lipids are the most commonly targeted. Several studies have shown that ROS can induce cellular damage by targeting the membranes through the peroxidation of polyunsaturated fatty acids and changing the membrane structure, the activity of cell membrane, lysosome, and mitochondria [4,5].

Like all cells, the renal cells have antioxidant defenses to compete with the severe oxidative harm after the IRI. When ROS are produced, phagocytes and macrophages are the main defense line for limiting inflammation [6].

Pro-inflammatory cytokines, such as Tumor Necrosis Factor-α (TNF-α), Interleukin-1 Beta (IL-1β), and Interleukin-6 (IL-6), have been found to enhance the ROS generation. Therefore, free radicals are critical for cytokine toxicity [7]. Cytokines are critical determinants of the immune responses and inflammatory reactions, and they are involved in many biological pathways [8,9,10,11]. Immune function deficiencies are prevalent in chronic renal failure. These abnormalities are caused by the decreased renal excretory action and uremic toxin accumulation, in addition to necrosis and apoptosis [12,13].

Extracellular signaling cascades, such as the pro-inflammatory markers IL-1β, TNF-α, and IL-6, activate macrophages that, in turn, stimulate various intracellular signaling pathways, including c-Jun N-terminal Kinase (JNK)/p38/Extracellular signal-regulated kinase (ERK) and the nuclear factor-kappaB (NF-κB) pathways in mammals [14,15]. NF-κB is a transcription factor activated and transferred to the nucleus to stimulate the expression of pro-inflammatory genes. It has been linked to several pathological processes and inflammatory diseases [16,17]. AKI management is needed to stop the production of pro-inflammatory markers including IL-1, IL-6, and TNF-α.

It is essential to develop new therapeutic modalities for the existed candidates [18,19,20,21,22]. A major source of novel agents with different pharmaceutical activities is herbal medicine, such as vincamine (VINCA) [23,24]. It is an indole alkaloid of clinical use against brain sclerosis and postoperative conditions [25]. It appears to act as an oxygen vector in living cells. Vincamine, particularly in the brain, has a selective vasoregulatory effect on the microcapillary circulation. This peripheral vasodilator action enhances the brain’s blood supply and is used as a nootropic agent to regulate aging signs [26]. It also acts as a brain metabolic stimulator by improving glucose utilization, adenosine triphosphate (ATP) generation, oxygen utilization, and offering better protection against hypoxia and cerebral ischemia. It improves serotonergic, noradrenergic, and dopaminergic functions possibly due to its antioxidant ability [27].

Moreover, synthetic agents, such as pantoprazole (PTZ), have shown various pharmacological effects [28,29,30,31]. PTZ was found to decrease the oxidative disequilibrium and toll-like receptor-4 protein expression in the renal tissues when given 30 min before the development of IRI [32].

In our previous study, we investigated the ameliorative action of pantoprazole against renal IRI on a molecular basis [33]. In this study, we explored, for the first time, the modulatory effect of vincamine, as a herbal candidate, on the activity of pantoprazole, as a synthetic drug, on treating the experimentally-induced renal IRI in rats and the potential mechanistic pathways underlying this effect by investigating MAPK (ERK1/2, JNK, p38)–NF-κB and apoptosis signaling pathways.

## 2. Results

### 2.1. Effect of Vincamine and/or Pantoprazole on Various Biochemical Markers

**Figure 1:**
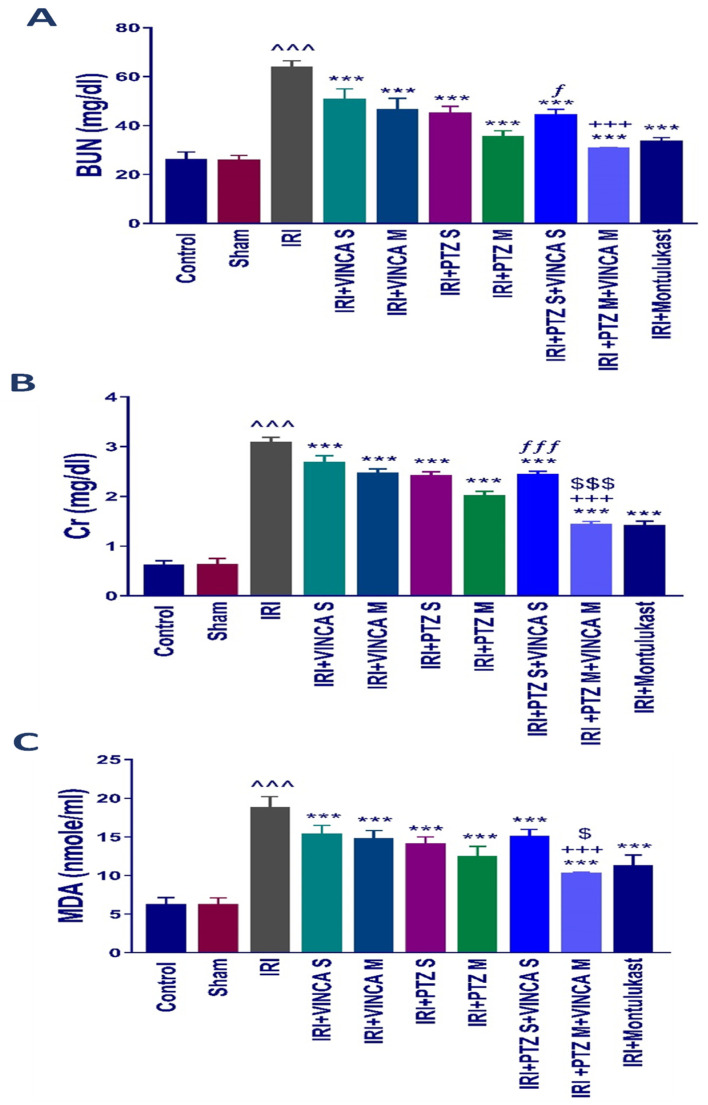
Effects of the different therapies on serum BUN (**A**), Scr (**B**), and MDA (**C**) levels. Bars represent mean ± SD. After the one-way analysis of variance (ANOVA) test, the Bonferroni post hoc test was used to determine the significant differences between the groups, where ^^^: *p* < 0.001, compared to the control rats. ***: *p* < 0.001, compared to the IRI group. +++: *p* < 0.001, compared to the IRI + VINCA M group. ƒ: *p* < 0.05 and ƒƒƒ: *p* < 0.001, compared to the IRI + VINCA S group. $: *p* < 0.05 and $$$: *p* < 0.001 compared to the IRI + PTZ M group.

Serum blood urea nitrogen (BUN) and serum creatinine (Scr), both markers of acute kidney damage, were estimated in the treated rats to understand the influence of vincamine and/or pantoprazole on the pathophysiology of IRI. The biochemical examination revealed a significant (*p* < 0.001) rise in BUN and Scr levels in the IRI rats compared to the control group (Figure 1A,B). In comparison to the IRI group, the IRI rats treated with a single dose of VINCA (VINCA S), multiple doses of VINCA (VINCA M), single dose of PTZ (PTZ S), multiple doses of PTZ (PTZ M), single doses of VINCA and PTZ (VINCA S + PTZ S), or multiple doses of PTZ and VINCA (PTZ M + VINCA M) had substantially lower BUN and Scr levels than the IRI rats (*p* < 0.001). Furthermore, compared to PTZ M, treating the IRI rats with and PTZ M + VINCA M resulted in a significant (*p* < 0.001) drop in Scr levels. Moreover, when compared to VINCA S, treating the IRI rats with VINCA S + PTZ S significantly (*p* < 0.05 and *p* < 0.001) decreased BUN and Scr levels, respectively. Finally, when compared to VINCA M, treating the IRI rats with VINCA M + PTZ M significantly (*p* < 0.001) decreased BUN and Scr levels.

By comparing the IRI rats to the normal rats, there was an obvious (*p* < 0.001) rise in the serum level of malondialdehyde (MDA), which is a lipid peroxidation by-product and an oxidative stress marker. In comparison to the IRI rats, VINCA S, VINCA M, PTZ S, PTZ M, VINCA S + PTZ S, and VINCA M + PTZ M treatments obviously (*p* < 0.001) reduced blood MDA levels. Moreover, treating the IRI rats with VINCA M + PTZ M resulted in a significant (*p* < 0.05 and *p* < 0.001) reduction in blood MDA levels when compared to PTZ M or VINCA M, respectively (Figure 1C). In these experiments, Montelukast was applied as a control therapy, which consistently exhibited protective benefits (Figure 1A–C) as previously described [34].

### 2.2. The Inflammatory Cytokines (TNF-α, IL-1β, and IL-6) Levels in Blood

**Figure 2:**
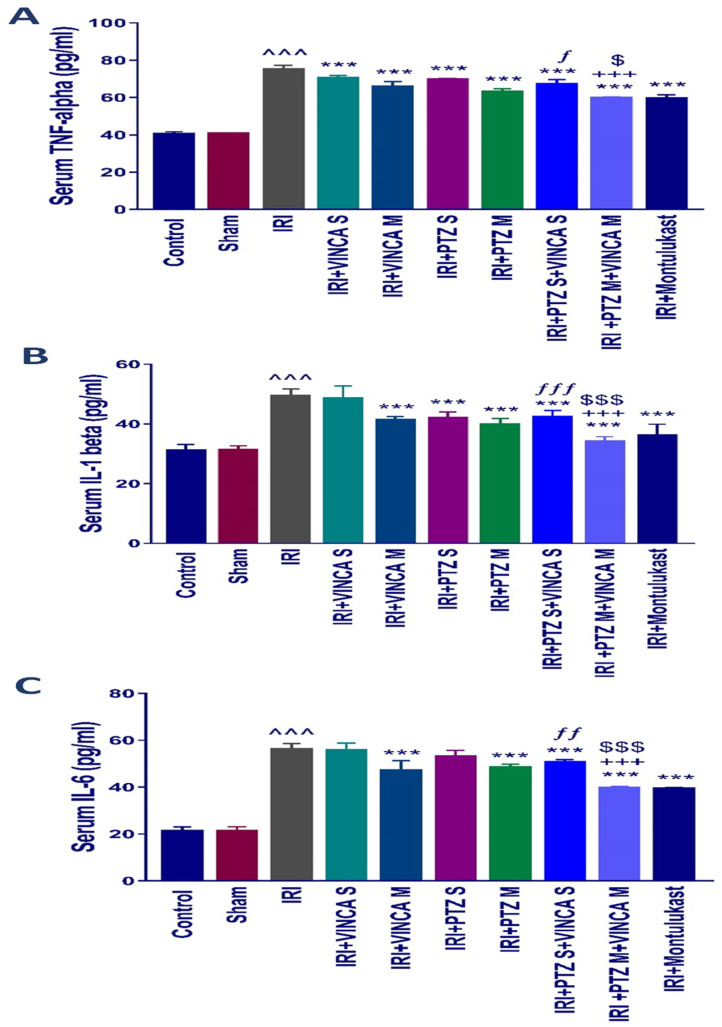
Impact on the inflammatory cytokine levels. Serum levels of (**A**) TNF-α, (**B**) IL-1β, and (**C**) IL-6 (pg/mL) for the studied groups. Bars represent mean ± SD. After the one-way ANOVA test, the Bonferroni post hoc test was used to determine the significant differences between the groups, where ^^^: *p* < 0.001, compared to the control rats. ***: *p* < 0.001, compared to the IRI rodents. $: *p* < 0.05 and $$$: *p* < 0.001, compared to the IRI + PTZ M group. ƒ: *p* < 0.05, ƒƒ: *p* < 0.01, and ƒƒƒ: *p* < 0.001, compared to the IRI + VINCA S group. +++: *p* < 0.001, compared to the IRI + VINCA M group.

The pro-inflammatory mediators, TNF-α, IL-1β, and IL-6, in the serum of the tested rats were evaluated to test if vincamine and/or pantoprazole had anti-inflammatory impacts. The detected pro-inflammatory cytokines TNF-α, IL-1β, and IL-6 in serum were significantly (*p* < 0.001) elevated in the IRI group when compared to the control rats as shown in (Figure 2A–C). When the IRI rats were given VINCA M, PTZ M, VINCA S + PTZ S or PTZ M + VINCA M the serum TNF-α, IL-1β and IL-6 levels significantly decreased (*p* < 0.001) in comparison to the IRI group.

In addition, VINCA M + PTZ M treatment significantly (*p* < 0.001) reduced serum TNF-α, IL-1β, and IL-6 levels compared to the VINCA M treatment. Finally, treating the IRI rats with VINCA M and PTZ M significantly (*p* < 0.05, *p* < 0.001, and *p* < 0.001) suppressed serum TNF-α, IL-1β, and IL-6 levels, respectively, compared to the PTZ M treatment.

### 2.3. B-Cell Lymphoma 2 (Bcl-2) and Bcl-2 Associated X-Protein (Bax) Genes Expression

**Figure 3:**
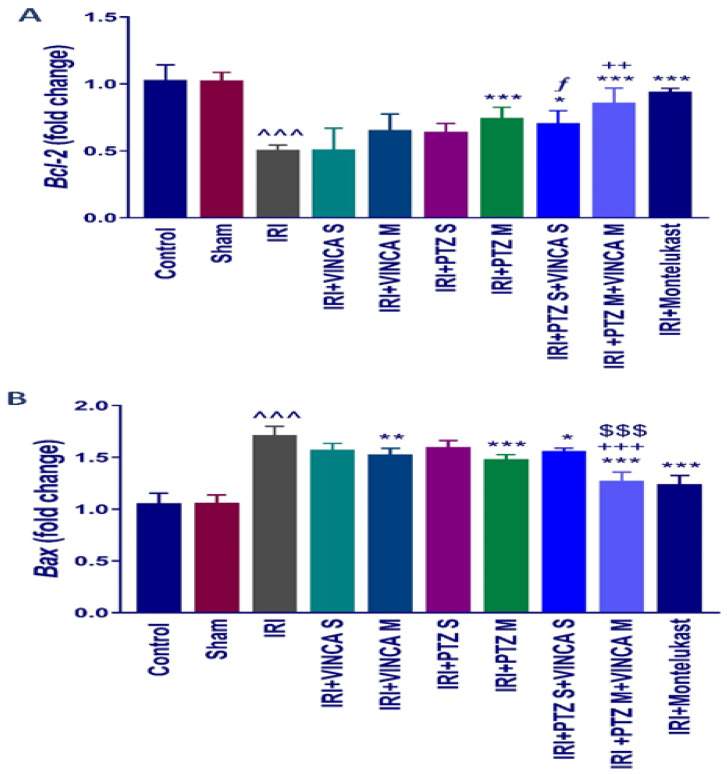
*Bcl-2* (**A**) and *Bax* (**B**) molecular expressions in the kidneys of the studied groups using quantitative real-time polymerase chain reaction (qRT-PCR). Data were presented as fold changes relative to the control group. Bars represent mean ± SD. After the one-way ANOVA test, the Bonferroni post hoc test was used to determine the significant differences between the groups, where ^^^: *p* < 0.001, compared to the control animals. *: *p* < 0.05, **: *p* < 0.01, and ***: *p* < 0.001, compared to the IRI group. $$$: *p* < 0.001, compared to the IRI + PTZ M group. ƒ: *p* < 0.05 compared to the IRI + VINCA S group. ++: *p* < 0.01 and +++: *p* < 0.001, compared to the IRI + VINCA M group.

To assess the influence of pantoprazole and/or vincamine on apoptosis, the expression of anti- and pro-apoptotic genes, *Bcl-2* and *Bax*, was examined in the studied groups. Figure 3A indicated that the IRI significantly (*p* < 0.001) reduced the renal mRNA levels of *Bcl-2* when compared to the control rats using *glyceraldehyde-3-phosphate dehydrogenase (GAPDH)* as a housekeeping gene. In addition, PTZ M, VINCA S + PTZ S, and VINCA M + PTZ M treatments significantly (*p* < 0.001, *p* < 0.05, and *p* < 0.001, respectively) elevated the *Bcl-2* gene expression. Furthermore, the VINCA S + PTZ S treatment significantly (*p* < 0.05) increased the renal mRNA levels of *Bcl-2* compared to the VINCA S-treated group. While VINCA M + PTZ M treatment significantly (*p* < 0.01) elevated the renal mRNA levels of *Bcl-2* compared to the VINCA M-treated group.

In addition, Figure 3B indicated an elevated *Bax* molecular expression (*p* < 0.001) in the IRI-induced rats compared to the control. VINCA M, PTZ M, VINCA S + PTZ S, and VINCA M + PTZ M treatments significantly (*p* < 0.01, *p* < 0.001, *p* < 0.05, and *p* < 0.001 respectively) inhibited the renal *Bax* gene expression compared to the IRI rats. In addition, VINCA M + PTZ M treatment significantly (*p* < 0.001) inhibited the renal *Bax* expression compared to the PTZ M or VINCA M treatment.

### 2.4. p-JNK1/2, p-ERK1/2, p-P38, Cleaved Caspase-3, and NF-kB Quantification in the Kidneys

Western blotting was used to determine the effect of vincamine and/or pantoprazole on the measured proteins after using montelukast as control and normalizing the intensities of the bands to β-actin. All the measured proteins were up-regulated in the IRI group relative to the control animals (*p* < 0.001). Up-regulation of phosphor-JNK1/2/total JNK1/2, phosphor-ERK1/2/total ERK1/2, phosphor-P38/total P38, cleaved caspase-3/caspase-3, and the total NF-kB proteins was observed in the injured rats compared to the normal rats.

**Figure 4:**
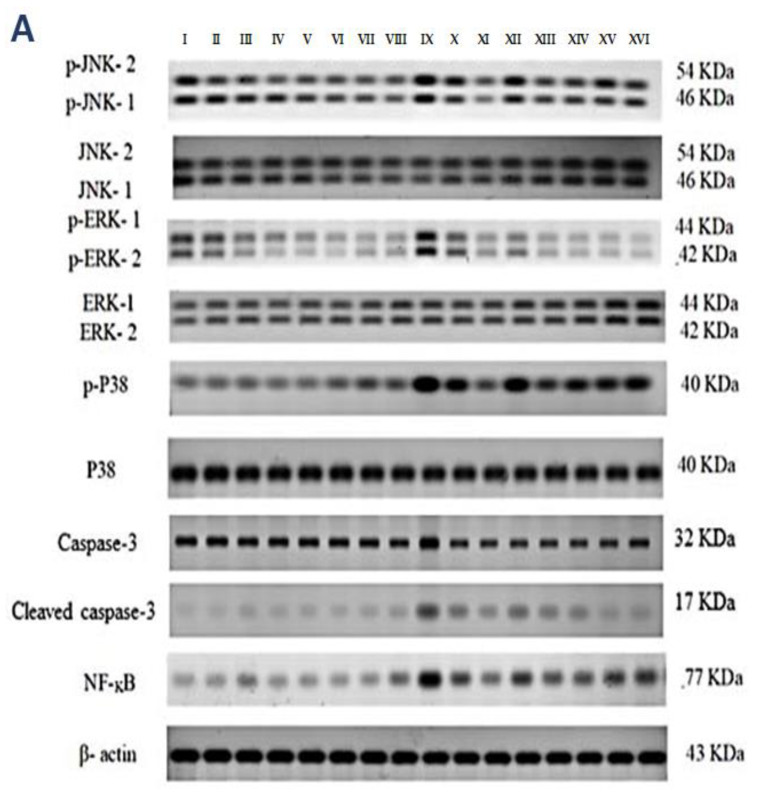

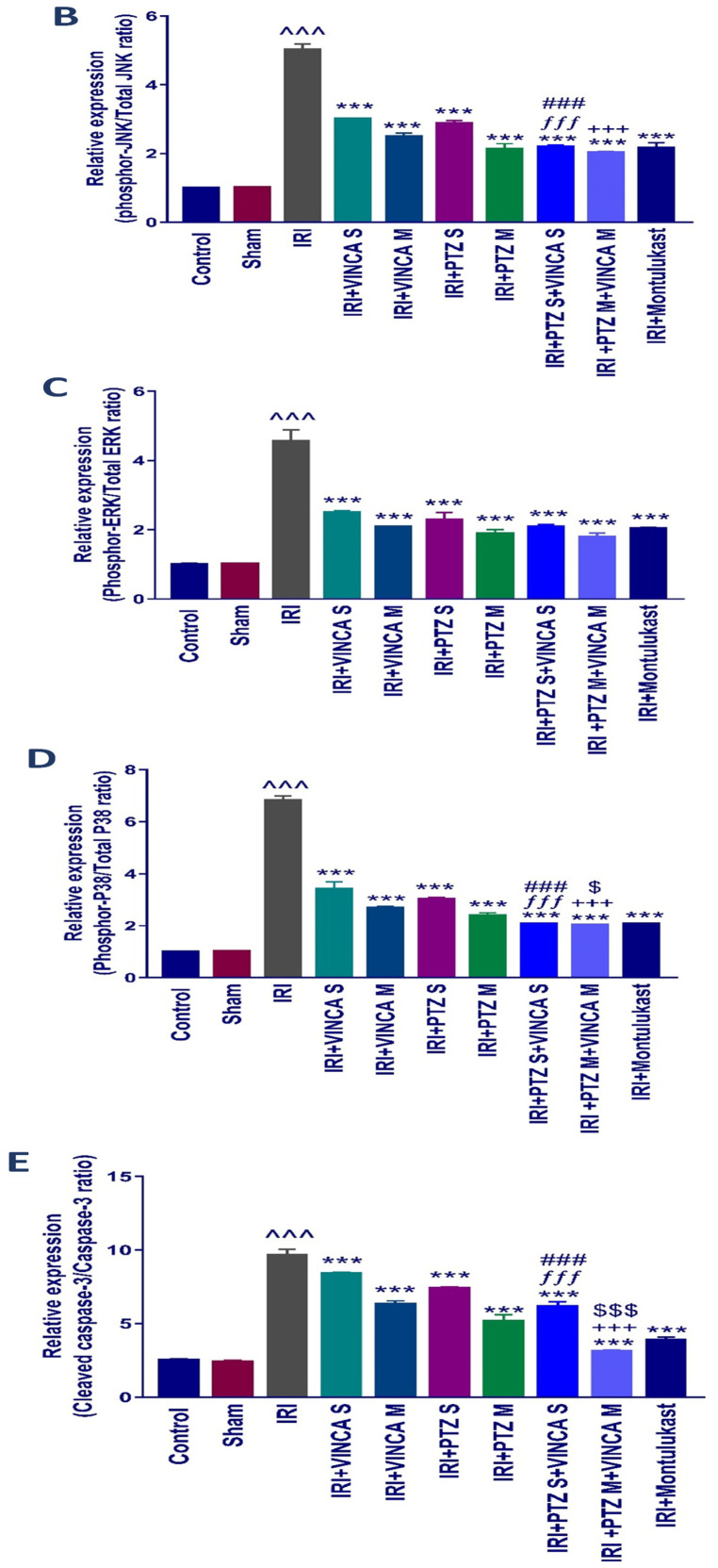

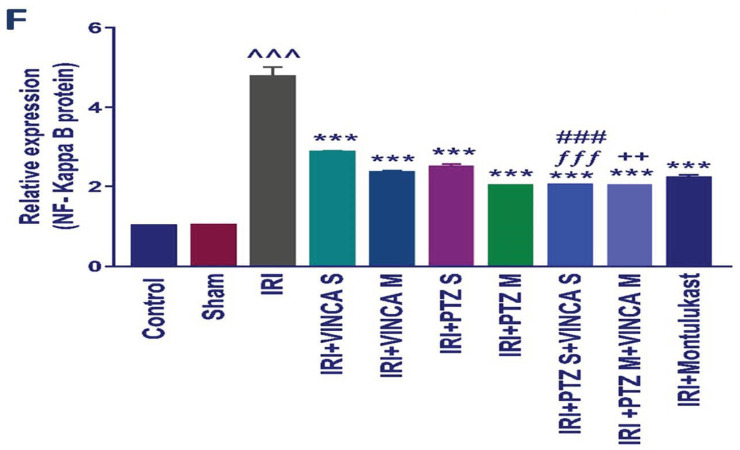
Effect on JNK1/2, ERK1/2, p38, caspase-3, and NF-kB proteins expression. (**A**) Western blot results of the measured proteins in kidneys of the studied groups. (**B**–**F**) Expressions of phosphor-JNK1/2/total JNK1/2, phosphor-ERK1/2/total ERK1/2, phosphor-P38/total P38, cleaved caspase-3/caspase-3, and the total NF-kB proteins, respectively. Bars represent mean ± SD. After the one-way ANOVA test, the Bonferroni post hoc test was used to determine the statistical significance between the studied groups, where ^^^: *p* < 0.001, compared to the control animals. ***: *p* < 0.001, compared to the injured animals. **###**: *p* < 0.001, compared to the IRI + PTZ S group. $: *p* < 0.05 and $$$: *p* < 0.001, compared to the IRI + PTZ M group. ƒƒƒ: *p* < 0.001 compared to the IRI + VINCA S group. ++: *p* < 0.01 and +++: *p* < 0.001, compared to the IRI + VINCA M group.

Intriguingly, the administration of VINCA M + PTZ M significantly reduced (*p* < 0.001) the five measured proteins when compared to the injured rats. Moreover, the combination of PTZ and VINCA had a better effect than the single treatments (Figure 4).

### 2.5. Histopathological Analysis of the Studied Groups

The impact of vincamine and/or pantoprazole was confirmed by histopathological analysis of the kidney tissue sections of the studied rats, with montelukast as a control therapy.

Groups I (Control), II (Sham), III (VINCA S), IV (VINCA M), V (PTZ S), VI (PTZ M), VII (VINCA S + PTZ S), and VIII (VINCA M + PTZ M) revealed no pathological changes and the usual histological composition of the cortex glomeruli and tubules were observed. On the other hand, marked infiltrations of inflammatory cells between the damaged tubules in the kidney cortex were seen in the injured rats (group IX). Moreover, at the corticomedullary section of the tubules, localized coagulative necrosis was seen in the lining epithelium.

**Figure 5:**
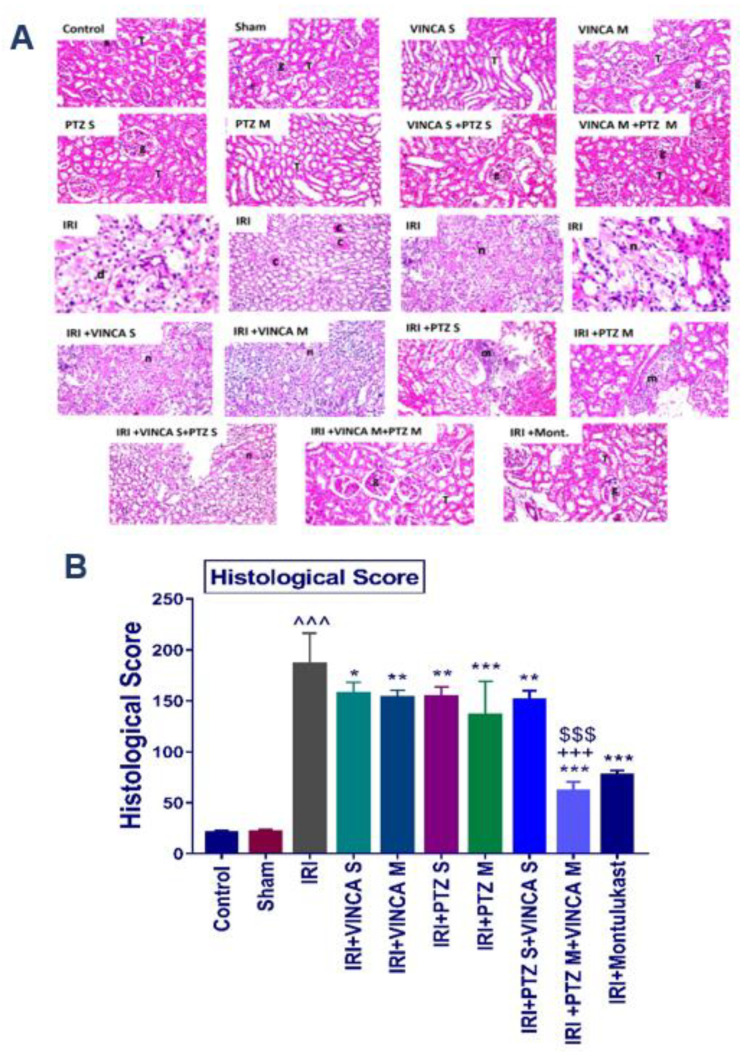
Histopathological analysis of the kidneys of the studied groups. (**A**) The hematoxylin and eosin (H & E) slides of the studied groups: the control, Sham, VINCA S, VINCA M, PTZ S, PTZ M, VINCA S + PTZ S, VINCA M + PTZ M, IRI, (IRI + VINCA S), (IRI + VINCA M), (IRI + PTZ S), (IRI + PTZ M), (IRI + VINCA S + PTZ S), (IRI + VINCA M + PTZ M), and (IRI + Montelukast) groups. With (g) representing the cortical glomeruli, (T) tubules, (m) periglomerular focal inflammatory cell aggregates, (d) degraded tubules, (c) eosinophilic casts, and (n) focal coagulative necrosis. Magnification factor: 40. (**B**) Histological scores of the damage. Bars represent mean ± SD. After the one-way ANOVA test, the Bonferroni post hoc test was used to determine the statistical significance between the studied groups, where ^^^: *p* < 0.001, compared to the control animals. *: *p* < 0.05, **: *p* < 0.01, and ***: *p* < 0.001, compared to the injured animals. $$$: *p* < 0.001, compared to the IRI + PTZ M group. +++: *p* < 0.001, compared to the IRI + VINCA M group.

Moreover, group X (IRI + VINCA S) displayed mild coagulative necrosis in some tubules at the cortex. Furthermore, in group XI (IRI + VINCA M), the corticomedullary and medullary portions showed mild focal coagulative necrosis in some tubules. In addition, group XII (IRI + PTZ S) showed moderate focal inflammatory cell aggregations surrounding the glomeruli. Group XIII (IRI + PTZ M) displayed mild focal perivascular inflammatory cells infiltrations surrounding the blood vessels at the cortex. Meanwhile, in group XIV (IRI + VINCA S+ PTZ S), the corticomedullary and medullary portions showed unremarkable focal coagulative necrosis in some tubules. More interestingly, in group XV (IRI + VINCA M + PTZ M) and group XVI (IRI + Montelukast), there were no histopathological alterations as shown in (Figure 5A, see the histological score B).

## 3. Discussion

The IRI is defined as the reduction of the blood flow to an organ accompanied by restoration of the flow of blood and oxygen. Inflammatory cascades, including ROS, cytokines release, and leukocyte stimulation, can happen as a result of infarction and intensify the tissue damage [33]. The IRI causes pathological conditions in the kidney termed AKI which is a clinical disorder with rapid renal failure and elevated mortality rates [35].

The current focus on herbal products inspires scientists to investigate natural agents in many disorders [36,37,38,39]. Many studies have shown that natural components can play an essential role in preventing human diseases [40,41,42,43]. Among them, vincamine has been reported to shield human corneal epithelial cells (HCECs) from lipopolysaccharides (LPS)-induced injuries. LPS significantly diminished the viability of HCECs, while the treatment with vincamine improved the viability of HCECs on a dose-dependent basis. Following the administration of LPS, the mRNA expression levels of *IL-6*, *IL-8*, *IL-1β*, and *TNF-α* in HCECs were substantially elevated indicating the activation of inflammatory reactions [44]. The reduction of these inflammatory factors by vincamine was also detected in a dose-dependent manner confirming its antioxidant efficacy through decreasing oxidative stress and inhibiting inflammation.

In this study, the IRI enhanced the formation of the lipid peroxidation by-product, MDA, as well as the levels of Scr and BUN, as previously reported [45,46]. On the other side, for the first time, the combination of VINCA and PTZ in multiple doses significantly reduced MDA, Scr, BUN levels, and improved the ischemic renal dysfunction more than VINCA alone or PTZ alone as single or multiple doses due to the synergistic effect of vincamine on pantoprazole. Furthermore, normal rats that were administered VINCA and/or PTZ showed no significant changes in all the studied parameters, indicating their safety; data were shown in the Supplementary Materials.

The IRI’s pathophysiology in the kidneys is very complicated, but specific pathological mechanisms, such as neutrophil activation, ROS generation, and release of inflammatory cytokines, are encountered. Research has shown the advantages of different therapies in the fight against IRI [47,48].

NF-κB is a transcription factor that controls the rate of gene expression of pro-inflammatory cytokines by binding to DNA regulatory regions in the cells. The NF-κB protein was up-regulated by IRI to increase the transcription of different inflammatory cytokines (TNF-α, IL-6, and IL-1β) [39]. The mitogen-activated protein kinase (MAPK) pathway, which includes ERK1/2, JNK1/2, and p38, is activated by the stimulation of macrophages. It represents an important component in regulating apoptosis and pro-inflammatory cytokines [49]. Furthermore, its stimulation has been linked to the increased NF-κB expression [41,42]. In this study, the levels of the pro-inflammatory cytokines, the expression of the apoptotic genes, *Bax* and *Bcl-2*, and the expression of ERK1/2, JNK1/2, p38, cleaved caspase-3, and NF-κB proteins were estimated.

In the present study, montelukast, a selective antagonist of cysteinyl leukotriene receptor 1, was used as a positive control because it was reported that montelukast protects kidney tissue in renal IRI in rats by regulating the generation of inflammatory cytokines and modulating the antioxidant status [50]. The current findings revealed that the combination of VINCA and PTZ in multiple doses reduced cytokines release by inhibiting the NF-κB protein expression, reducing the phosphorylation of p38, JNK1/2, and ERK1/2 proteins, regulating the MAPK (ERK1/2, JNK1/2, p38) signaling pathway, and reducing the ischemic injury and apoptosis more effectively than VINCA or PTZ alone as single or multiple doses.

Previous studies showed that the overproduction of ROS and stimulation of MAPK and NF-κB led to the up-regulation of *Bax* and down-regulation of *Bcl-2* expressions, resulting in apoptosis [51,52,53].

In AKI, the pro-apoptotic *Bax* gene was substantially overexpressed compared to the anti-apoptotic *Bcl-2* gene in this study. By contrast, the *Bax* gene was down-regulated and the relative expression of *Bcl-2* was significantly up-regulated after the treatment with VINCA and PTZ combination in multiple doses more than VINCA alone or PTZ alone as single or multiple doses. Caspases-3, one of the primary apoptosis signaling molecules, signals cell death. The cleavage of procaspase occurs when the apoptotic cascade is activated. As a result, cleaved caspase-3 is a reliable apoptosis biomarker [45,46]. The ratio of cleaved caspase-3/caspase-3 proteins was elevated in the IRI rats.

Taken together, we demonstrated that the VINCA and PTZ combination in multiple doses mitigated the renal ischemic damage and limited apoptosis by suppressing *Bax*/*Bcl-2* genes expression and the ratio of cleaved caspase-3/caspase-3 proteins. Still, future work is required for more understanding the molecular mechanisms by which vincamine might exert its beneficial renal protection and further investigations are necessary, using different doses of vincamine, to increase the knowledge concerning the ability of our findings to be clinically viable.

## 4. Materials and Methods

### 4.1. Drugs and Chemicals

All the chemicals were purchased from commercial suppliers and were of analytical quality. Pantoprazole sodium (98 percent, Taketa GmbH, Byk-Gulden-Str. 2, Konstanz, Baden-Württemberg, Germany) was formulated at a concentration of 40 mg/mL in 0.9 percent sodium chloride injection solution [54] and kept at 4 °C in dark. Vincamine powder (October Pharmaceutical Company, Giza, Egypt) was freshly reconstituted in a vehicle (1 percent carboxymethyl cellulose) at 30 mg/mL and kept at 4 °C in dark. Montelukast powder (Sedico Pharmaceutical Company, Giza, Egypt) was freshly reconstituted in 0.9 percent sodium chloride injection solution at a concentration of 10 mg/mL.

### 4.2. Animals and Care

One-hundred-and-twenty-eight male Wistar albino rats (about 250–320 g, 6–10 weeks old) were kept in cages with unlimited access to food and water. Rats were subdivided into sixteen groups (eight rats each). The study methods and animal care were conducted in accordance with the guidelines outlined by the Minia University’s Research Ethics Committee. The renal IRI was performed as previously described [33]. Rats were anesthetized by intraperitoneal (I.P.) injection of xylazine hydrochloride (10 mg/kg) and ketamine (50 mg/kg). Bilateral occlusion of the renal pedicles was performed using atraumatic microvascular clamps for 45 min. At the end of the ischemic period, the clips were removed to enable blood reperfusion. After the clamps were removed, the kidneys were examined for 1 min to preserve blood supply as shown by the return to their original color. Then, the abdomen was closed with moist sterile pad and surgical forceps.

Sham-operated rats underwent the same surgical procedures, except that the atraumatic clamps were not implemented. After that, the rats were starved for 12 h and allowed access only to water before scarification. For the biochemical analysis, sera were collected from the hearts and kept at −20 °C. Kidneys were rapidly dissected, washed with ice-cold phosphate-buffered saline (PBS, 0.1 M, pH 7.4), dried, and weighed.

The renal tissues were divided into three parts. The first portion was kept for western blotting as described before [55,56]. The second portion was kept in RNA latter, stored at 4 °C for 24 h, and then at −20 °C for the extraction of total RNA by the Trizol Reagent. The third portion was put in formalin 10% for the histopathological staining.

### 4.3. Experimental Design

Rats were subdivided into sixteen groups (eight rats each) and each group was treated as described in Table 1.

### 4.4. Biochemical Assay

The renal functions were assessed by determining Scr levels using (human diagnostic, Wiesbaden, Germany #10051) kits and serum BUN levels using (human diagnostic, Wiesbaden, Germany #10505) kits. Serum MDA levels were measured using (Biodiagnositic, Giza, Egypt # CAT. No. MD 2529) kits. All of these biochemical parameters were spectrophotometrically assessed following the manufacturer’s instructions.

### 4.5. Measurement of the Inflammatory Cytokines

TNF-α (Cat. No.: E-EL-R001996T), IL-1β (Cat. No.: E-EL-R001296T), and IL-6 (Cat. No.: E-EL-R001596T) serum levels were quantified using the ELISA kits (Elabscience^®^, Houston, TX, USA) and following the manufacturer’s instructions.

### 4.6. Quantitative Real-Time Polymerase Chain Reaction (qRT-PCR)

A digital homogenizer (Branson Digital homogenizer^®^, Danbury, CT, USA) was used to homogenize 100 mg of the kidney tissue in 1 ml of TRIzol TM reagent (Amresco, Solon, OH, USA). The TRIzol TM RNA Extraction Reagent (Amresco, Solon, OH, USA) was used to extract RNA from the biopsy specimens according to the manufacturer’s instructions. The RevertAid H Minus First Strand cDNA Synthesis Kits (#K1632, Thermo Science Fermentas, St. Leon-Ro, Germany) were used to synthesize cDNA for comparable quantities of the total RNA in all samples following the manufacturer’s instructions. Using the reverse transcription product as a template, the RT-PCR was performed on the Applied Biosystems Step One Plus thermal cycler. NCBI primer blast designed the primers which were then manufactured by Invitrogen [33]. Data were analyzed according to the 2^−ΔΔct^ method using the *GAPDH* gene as the reference gene. The used *BAX* gene primers were sense: 5′- GGT GTT GAC GGT TCA CTT GC -3′and antisense: 5′- AAC GCC TGG ATG GGC TTT TA -3′. The used *Bcl-2* gene primers were sense: 5′- TGT ATC AAA CCA TGC GGC TG -3′ and antisense: 5′- GGC TGG TTT TAC CGC ACC TT -3′. The used *GAPDH* gene primers were sense: 5′- ACC AAC TGC TTA GCC CCC C -3′ and antisense: 5′- GCA TGT CAG ATC CAC AAC GG -3′.

### 4.7. Western Blotting Analysis

A digital homogenizer (Branson Digital homogenizer ^®^, Danbury, CT, USA) was utilized to homogenize the kidney tissues using a protein extraction buffer. Protein contents of the supernatants were estimated by protein assay kits (Quant-iT™ Protein Assay Kit, Carlsbad, CA, USA). The protein content of 50 µg of every homogenate was deposited to a membrane of PVDF following electrophoresis on a 12.5 percent sodium dodecyl sulfate-polyacrylamide gel (EMD Millipore, Billerica, MA, USA). The estimated proteins were p-38 (1:1000, Abcam, Cambridge, UK, #Ab31828), phospho-p-38 (1:1000, Abcam, # ab207483), phosphor-ERK (1:1000, Abcam, Cambridge, UK, #Ab207470), ERK (1:1000, Abcam, Cambridge, UK, #ab176660), NF-kB p65 (1:5000, Abcam, Cambridge, UK, #Ab32536), JNK (1:1000, Abcam, Cambridge, UK, #Ab179461), p-JNK (1:100, Abcam, Cambridge, UK, #Ab207477), cleaved caspase-3 (1:1000, Abcam, Cambridge, UK, #Ab214430), caspase-3 (1:1000, Abcam, Cambridge, UK, #Ab184787), and β-actin (sc-1615, 1:250) (Abcam, Cambridge, UK, #Ab8226). After washing, the membranes were incubated for one hour with the secondary antibodies. Using the ECL detection technique, the protein bands were seen through chemiluminescence (Amersham Bioscience, Freiburg, Germany). Densitometry was used to quantify the band intensity to β-actin bands using the Gel-Pro Analysis 7.0 program (Media Cybernetics, Rockville, MD, USA).

### 4.8. Histological Assessment

Kidney specimens were taken from the groups, fixed in 10 percent formalin for twenty-four hours, irrigated with tap water, and dehydrated with serial alcohol dilutions ranging from 10% to 100% (ethyl and absolute ethyl). Xylene washes and paraffin fixation at 56 °C for one day were performed. At a thickness of 4 microns, the paraffin blocks were sectioned with a sliding microtome. The acquired tissue pieces were stained with H & E for the usual examination using a light microscope (Olympus BH 2, Tokyo, Japan) [58]. Each kidney had 100 assessed intersections and each tubular profile was given a score ranging from 0 to 3:0, normal histology; 1, tubular cells swelling, brush border loss, and nuclear condensation with up to one-third the tubular profile showing nuclear loss; 2, as the score 1 with greater than one-third and less than two-thirds of the tubular profile showing nuclear loss; 3, more than two-thirds the tubular profile shows nuclear loss. The overall score for each kidney was determined by adding all the 100 scores up to a maximum of 300. All the histology investigations were done in a blinded manner [59].

### 4.9. Statistical Analysis

Graph Pad Prism version 7 statistical software was used to tabulate the data (GraphPad, La Jolla, CA, USA). The one-way analysis of variance (ANOVA) test followed by the Bonferroni post hoc test for multiple comparisons was used to assess statistical differences between the groups. Statistical significance is defined as a *p*-value of less than or equal to 0.05.

## 5. Conclusions

For the first time, our study showed that the strong anti-inflammatory and anti-apoptotic actions of VINCA, in combination with PTZ in multiple dosages, are accountable for the mitigation of the IRI-induced kidney damage. It attenuated apoptosis via modulating the expression of *Bax* and *Bcl-2* genes, as well as the cleaved caspase-3 protein. It also inhibited the extracellular signaling pathways such as the pro-inflammatory cytokines (TNF-α, IL-1β, and IL-6) and suppressed the intracellular signaling pathways, such as MAPK (ERK1/2, JNK, p38) and NF-κB. Vincamine, as a herbal candidate, enhanced and modulated the activity of pantoprazole on treating renal IRI.

## Figures and Tables

**Table 1:** The designed groups and their treatment.

Group I (Control):	Normal control rats received 0.2 mL saline (i.p.) injection for 10 days.
Group II (Sham group):	Rats were given a fake operation and received 0.2 mL saline (i.p.) injection for 10 days.
Group III (VINCA S):	Sham-treated rats received a single dose of VINCA (120 mg base/kg in a vehicle (1% carboxymethyl cellulose)) orally [34].
Group IV (VINCA M):	Sham-treated rats received multiple oral doses of VINCA (30 mg base/kg) once daily for 6 days [34].
Group V (PTZ S):	Sham-treated rats received a single dose of PTZ (160 mg/kg, i.p.) [57].
Group VI (PTZ M):	Sham-treated rats received multiple doses of PTZ (40 mg/kg, orally) twice daily for 10 days [57].
Group VII (VINCA S + PTZ S):	Sham-treated rats received a single dose of VINCA (120 mg base/kg) orally [34], and a single dose of PTZ (160 mg/kg) by (i.p.) injection [57].
Group VIII (VINCA M + PTZ M):	Sham-treated rats received multiple doses of VINCA (30 mg base/kg, orally) once daily for 6 days [34], together with multiple doses of PTZ (40 mg/kg, orally) twice daily for 10 days [57].
Group IX (IRI):	IRI rats received 0.2 mL saline by (i.p.) for 10 days.
Group X (IRI + VINCA S):	IRI treated rats received a single dose of VINCA (120 mg base/kg, orally) [34].
Group XI (IRI + VINCA M):	IRI treated rats received multiple doses of VINCA (30 mg base/kg) once daily for 6 days orally [34].
Group XII (IRI + PTZ S):	IRI treated rats received a single dose of PTZ (160 mg/kg, i.p.) [57].
Group XIII (IRI + PTZ M):	IRI treated rats received multiple doses of PTZ (40 mg/kg, orally) twice daily for 10 days [57].
Group XIV (IRI + VINCA S + PTZ S):	IRI treated rats received single dose of VINCA (120 mg base/kg) orally [34], together with a single dose of PTZ (160 mg/kg, i.p.) [57].
Group XV (IRI + VINCA M + PTZ M):	IRI treated rats received multiple oral doses of VINCA (30 mg base/kg) once daily for 6 days [34], together with multiple oral doses of PTZ (40 mg/kg) twice daily for 10 days [57].
Group XVI (IRI + Montelukast):	IRI treated rats were received single dose of Montelukast (10 mg/kg, i.p.) [50], used as a positive control group.

## Data Availability

All data are fully available and included in the manuscript.

## Supplementary Materials

The following are available online, Figure S1. Effects of the different therapies on serum BUN (**A**), Scr (**B**), and MDA (**C**) levels; Figure S2. Impact on the inflammatory cytokines levels. Serum levels of (**A**) TNF-α, (**B**) IL-1β, and (**C**) IL-6 (pg/ml) for the studied groups; Figure S3. *Bcl-2* (**A**) and *Bax* (**B**) molecular expressions in the kidneys of the studied groups using RT-PCR; Figure S4. Effects on JNK1/2, ERK1/2, p38, caspase-3 and NF-kB proteins expression. (**B**–**F**) Expressions of phosphor-JNK1/2/total JNK1/2, phosphor-ERK1/2/total ERK1/2, phosphor-P38/total P38, cleaved caspase3/caspase-3 and total NF-kB proteins, respectively. Figure S5 (**B**). Histological score regarding the damage of the studied groups: Control, Sham, VINCA S, VINCA M, PTZ S, PTZ M, VINCA S + PTZ S, VINCA M + PTZ M, IRI, (IRI + VINCA S), (IRI + VINCA M), (IRI + PTZ S), (IRI + PTZ M), (IRI + VINCA S + PTZ S), (IRI+VINCA M + PTZ M), (IRI + Montelukast) (IRI + Mont.) groups.

## Sample Availability

Samples of the compounds are not available from the authors.
