# Supplementary material for: Vincamine Modulates the Effect of Pantoprazole in Renal Ischemia/Reperfusion Injury by Attenuating MAPK and Apoptosis Signaling Pathways
Source: Molecules. 2022 Feb 18;27(4):1383. doi: 10.3390/molecules27041383 (PMC8879001; doi:10.3390/molecules27041383)
Supplement: Supplementary file 1 [file molecules-27-01383-s001.zip › molecules-1579298-supplementary.pdf]

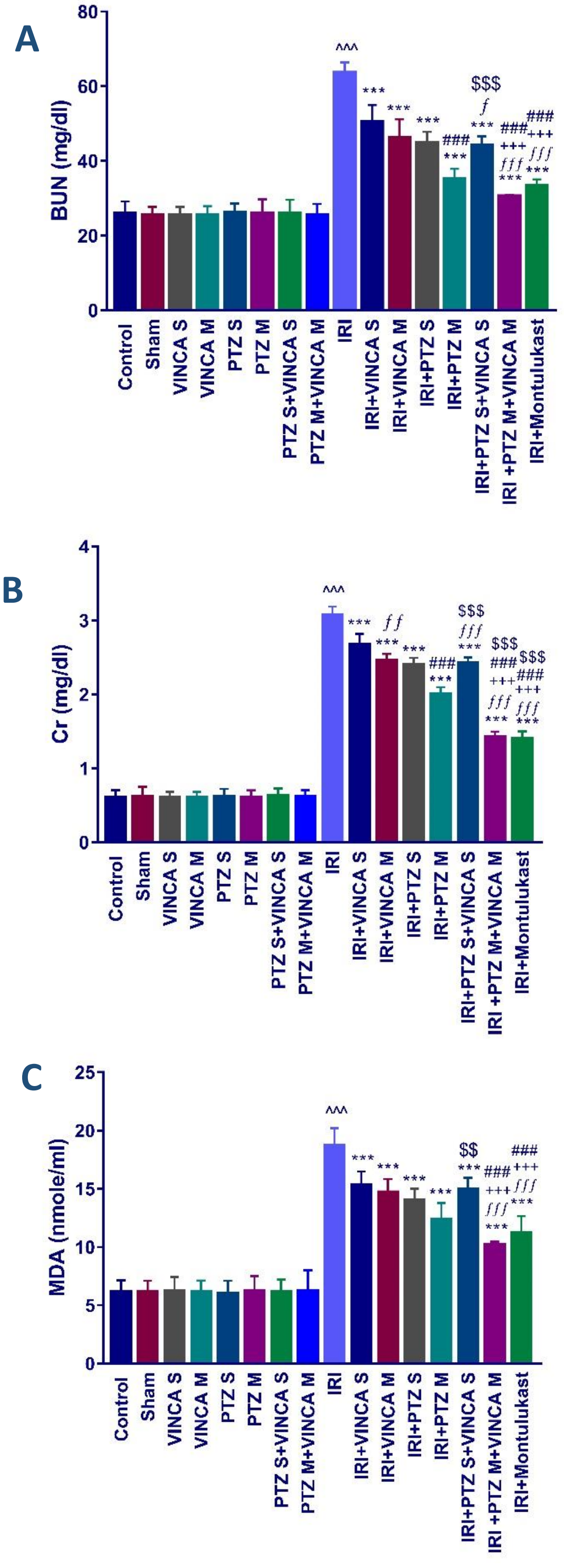
A
BUN (mg/dl)
0
20
40
60
80
B
Cr (mg/dl)
0
1
2
3
4
C
MDA (nmole/ml)
0
5
10
15
20
25
Control
Sham
VINCA S
VINCA M
PTZ S
PTZ M
PTZ S+VINCA S
PTZ M+VINCA M
IRI
IRI+VINCA S
IRI+VINCA M
IRI+PTZ S
IRI+PTZ M
IRI+PTZ S+VINCA S
IRI +PTZ M+VINCA M
IRI+Montulukast

Figure S1. Effects of the different therapies on serum BUN (A), Scr (B), and MDA (C) levels. Bars represent mean ± SD. After the one-way ANOVA test, the Bonferroni post-hoc test was used to determine the significant differences between the groups, where: ^^^: $P < 0.001$, compared to the control rats. ***: $P < 0.001$, compared to the IRI group. ###: $P < 0.001$, compared to the IRI + PTZ S group. +++: $P < 0.001$, compared to the IRI + VINCA M group. ƒ: $P < 0.05$, ƒƒ: $P < 0.01$, and ƒƒƒ: $P < 0.001$, compared to the IRI + VINCA S group. $: $P < 0.05$, $$: $P < 0.01$, $$$: $P < 0.001$ compared to the IRI + PTZ M group.

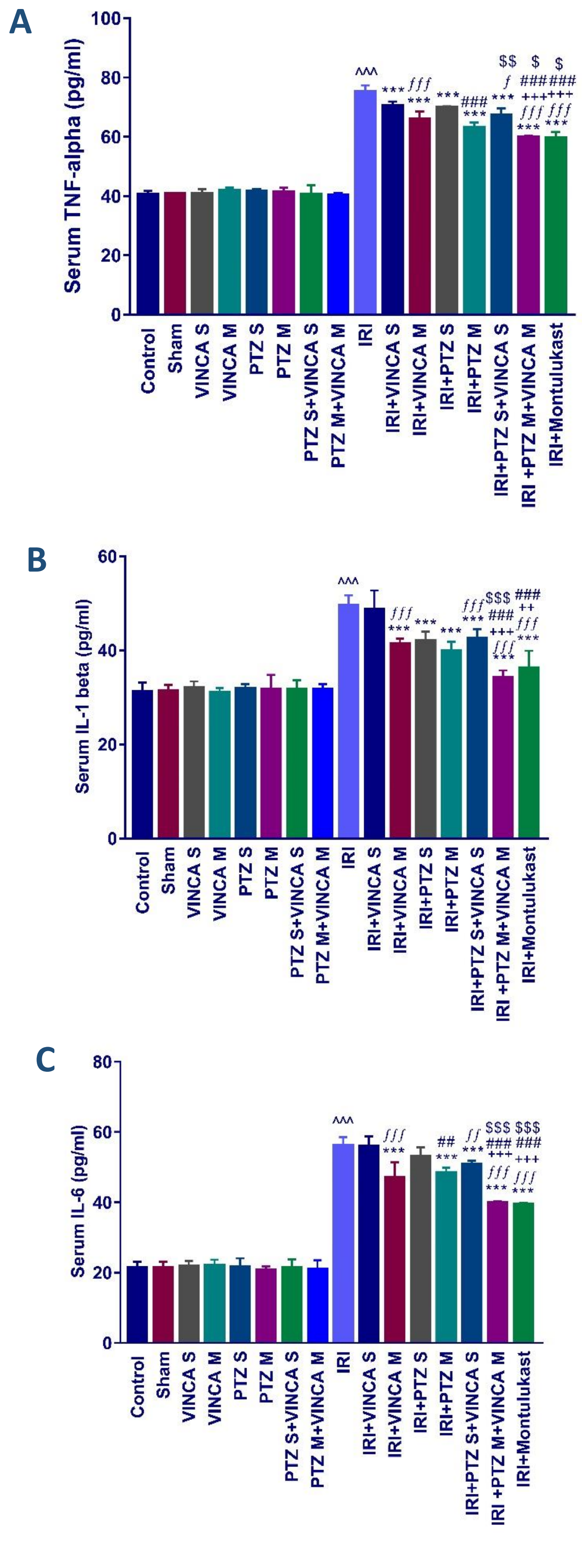
A
Serum TNF-alpha (pg/ml)
B
Serum IL-1 beta (pg/ml)
C
Serum IL-6 (pg/ml)
Control
Sham
VINCA S
VINCA M
PTZ S
PTZ M
PTZ S+VINCA S
PTZ M+VINCA M
IRI
IRI+VINCA S
IRI+VINCA M
IRI+PTZ S
IRI+PTZ M
IRI+PTZ S+VINCA S
IRI +PTZ M+VINCA M
IRI+Montulukast

Figure S2. Impact on the inflammatory cytokines levels. Serum levels of (A) TNF-α, (B) IL-1β, and (C) IL-6 (pg/ml) for the studied groups. Bars represent mean ± SD. After the one-way ANOVA test, the Bonferroni post-hoc test was used to determine the significant differences between the groups, where: ^^^: $P < 0.001$, compared to the control rats. ***: $P < 0.001$, compared to the IRI rodents. ##: $P < 0.01$, ###: $P < 0.001$, compared to the IRI + PTZ S group. $: $P < 0.05$, $$: $P < 0.01$, $$$: $P < 0.001$, compared to the IRI + PTZ M group. ƒ: $P < 0.05$, ƒƒ: $P < 0.01$, and ƒƒƒ: $P < 0.001$, compared to the IRI + VINCA S group. +++: $P < 0.001$, compared to the IRI + VINCA M group.

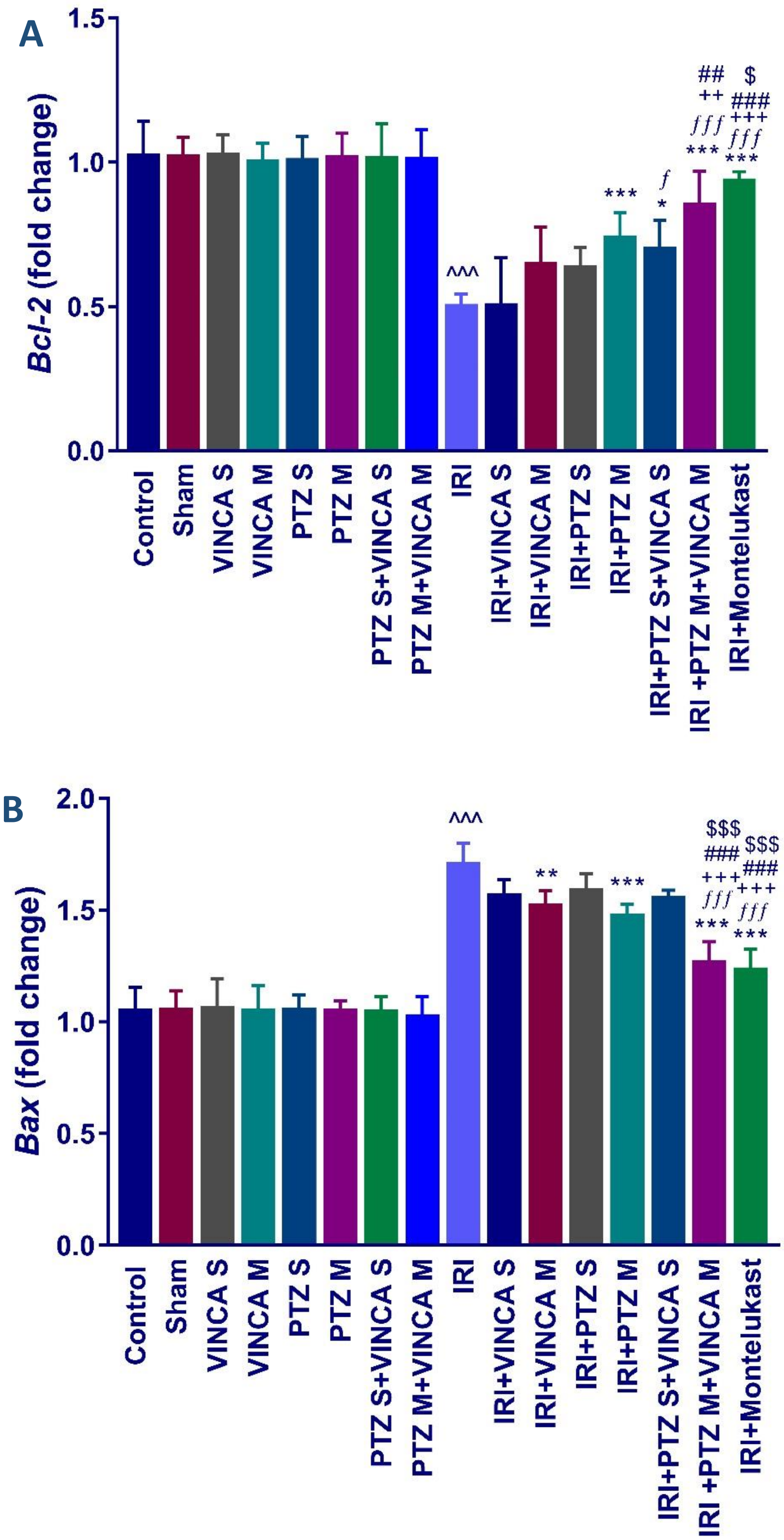
A
Bcl-2 (fold change)
0.0
0.5
1.0
1.5
Control
Sham
VINCA S
VINCA M
PTZ S
PTZ M
PTZ S+VINCA S
PTZ M+VINCA M
IRI
IRI+VINCA S
IRI+VINCA M
IRI+PTZ S
IRI+PTZ M
IRI+PTZ S+VINCA S
IRI +PTZ M+VINCA M
IRI+Montelukast
B
Bax (fold change)
0.0
0.5
1.0
1.5
2.0

Figure S3. *Bcl-2* (A) and *Bax* (B) molecular expressions in the kidneys of the studied groups using RT-PCR. Data were presented as fold changes relative to the control group. Bars represent mean ± SD. After the one-way ANOVA test, the Bonferroni post-hoc test was used to determine the significant differences between the groups, where: ^^^: $P < 0.001$, compared to the control animals. *: $P < 0.05$, **: $P < 0.01$, and ***: $P < 0.001$, compared to the IRI group. ##: $P < 0.01$, and ###: $P < 0.001$, compared to the IRI + PTZ S group. $: $P < 0.05$, compared to the IRI + PTZ M group. ++: $P < 0.01$, and +++: $P < 0.001$, compared to the IRI + VINCA M group. ƒ: $P < 0.05$, and ƒƒƒ: $P < 0.001$ compared to the IRI + VINCA S group.

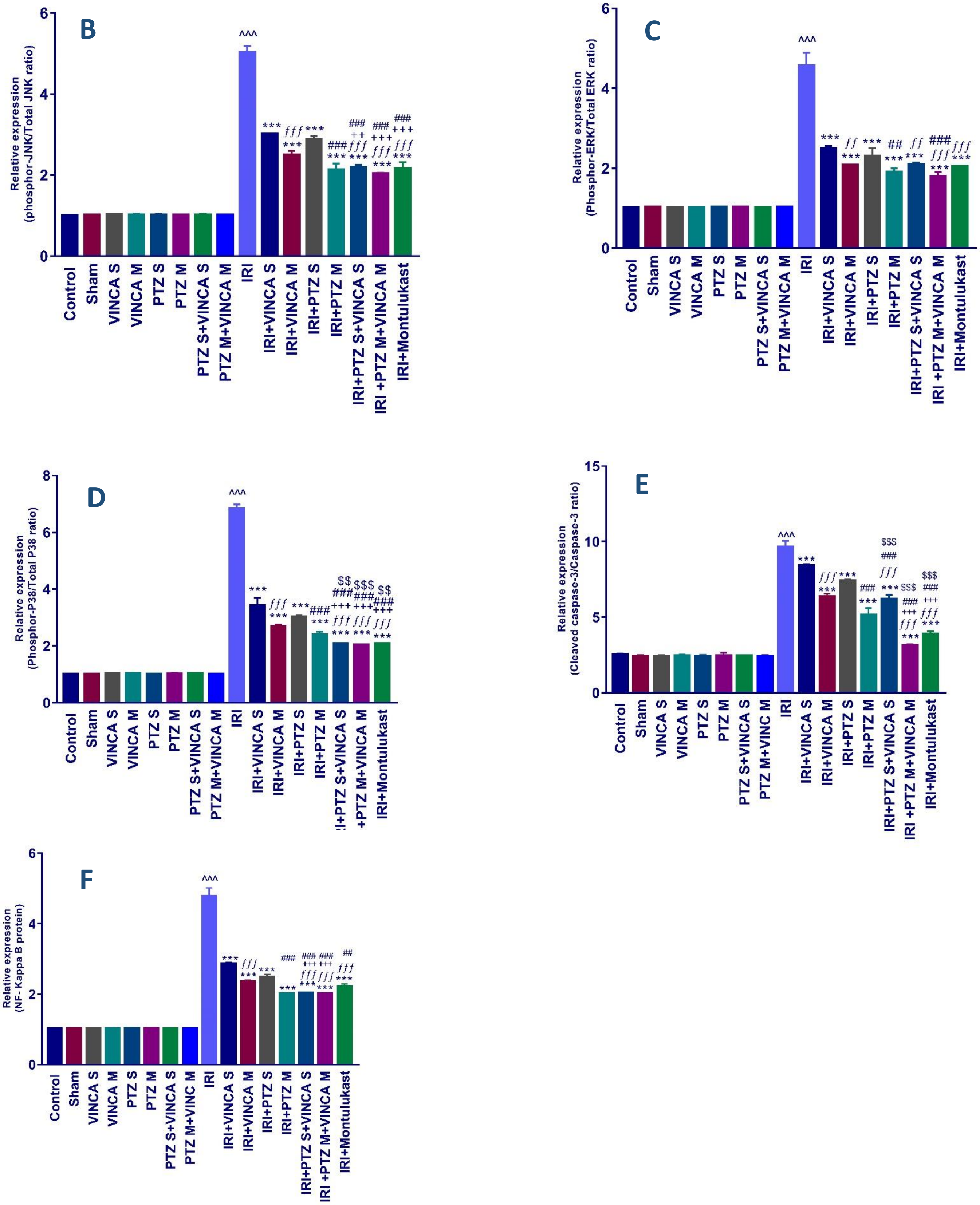
B
Relative expression
(phosphor-JNK/Total JNK ratio)
C
Relative expression
(Phosphor-ERK/Total ERK ratio)
D
Relative expression
(Phosphor-P38/Total P38 ratio)
E
Relative expression
(Cleaved caspase-3/Caspase-3 ratio)
F
Relative expression
(NF- Kappa B protein)
Control
Sham
VINCA S
VINCA M
PTZ S
PTZ M
PTZ S+VINCA S
PTZ M+VINCA M
PTZ M+VINC M
IRI
IRI+VINCA S
IRI+VINCA M
IRI+PTZ S
IRI+PTZ M
IRI+PTZ S+VINCA S
IRI +PTZ M+VINCA M
IRI+Montulukast

Figure S4. Effects on JNK1/2, ERK1/2, p38, caspase-3 and NF-kB proteins expression. (B, C, D, E, F) Expressions of phosphor-JNK1/2/total JNK1/2, phosphor-ERK1/2/total ERK1/2, phosphor-P38/total P38, cleaved caspase3/caspase-3 and total NF-kB proteins, respectively. Bars represent mean ± SD. After the one-way ANOVA test, the Bonferroni post-hoc test was used to determine the statistical significance between the studied groups, where: ^^^: P < 0.001, compared to the control animals. ***: P < 0.001, compared to the injured animals. ##: P < 0.01, and ###: P < 0.001, compared to the IRI + PTZ S group. $: P < 0.05, $$: P < 0.01, and $$$: P < 0.001, compared to the IRI + PTZ M group. ƒ: P < 0.05, and ƒƒƒ: P < 0.001 compared to the IRI + VINCA S group. +: P < 0.05, ++: P < 0.01, and +++: P < 0.001, compared to the IRI + VINCA M group.

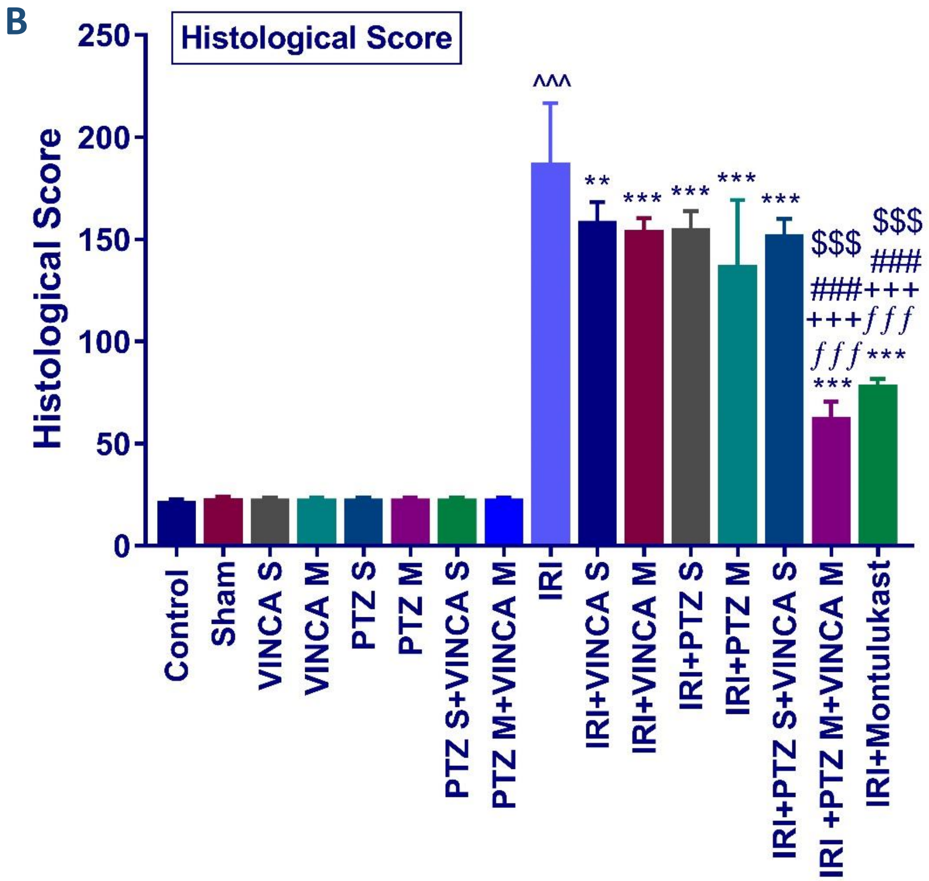

B
Histological Score
Histological Score
250
200
150
100
50
0
^^^
**
***
***
***
***
$$$
###
+++
ƒƒƒ
***
$$$
###
+++
ƒƒƒ
***
Control
Sham
VINCA S
VINCA M
PTZ S
PTZ M
PTZ S+VINCA S
PTZ M+VINCA M
IRI
IRI+VINCA S
IRI+VINCA M
IRI+PTZ S
IRI+PTZ M
IRI+PTZ S+VINCA S
IRI +PTZ M+VINCA M
IRI+Montulukast

Figure S5 (B). Histological score regarding the damage of the studied groups: Control, Sham, VINCA S, VINCA M, PTZ S, PTZ M, VINCA S + PTZ S, VINCA M + PTZ M, IRI, (IRI + VINCA S), (IRI + VINCA M), (IRI + PTZ S), (IRI + PTZ M), (IRI + VINCA S + PTZ S), (IRI+VINCA M + PTZ M), (IRI + Montelukast) (IRI + Mont.) groups. Bars represent mean ± SD. After the one-way ANOVA test, the Bonferroni post-hoc test was used to determine the statistical significance between the studied groups, where: ^^^: $P < 0.001$, compared to the control animals. *: $P < 0.05$, **: $P < 0.01$, and ***: $P < 0.001$, compared to the injured animals. ###: $P < 0.001$, compared to the IRI + PTZ S group. $$$: $P < 0.001$, compared to the IRI + PTZ M group. ƒƒƒ: $P < 0.001$ compared to the IRI + VINCA S group. +++: $P < 0.001$, compared to the IRI + VINCA M group.
